# Restlessness and an Increased Urge to Move (Drive for Activity) in Anorexia Nervosa May Strengthen Personal Motivation to Maintain Caloric Restriction and May Augment Body Awareness and Proprioception: A Lesson From Leptin Administration in Anorexia Nervosa

**DOI:** 10.3389/fpsyg.2022.885274

**Published:** 2022-07-25

**Authors:** Regina C. Casper

**Affiliations:** Department of Psychiatry and Behavioral Sciences, Stanford University, Stanford, CA, United States

**Keywords:** restlessness, drive for activity, leptin, body image, self-image, anorexia nervosa

## Abstract

Anorexia nervosa (AN), a disorder of voluntary food restriction leading to severe weight loss in female adolescents, remains an enigma. In particular, the appropriation of the starved thin body into the self-concept in AN is a process insufficiently researched and still poorly understood. Healthy humans undergoing starvation experience a slowing of movements and avoid voluntary exercise. By contrast, AN tends to be not infrequently associated with voluntary, sometimes excessive and/or compulsive exercise. Such deliberate exercise, not reported in starvation, seems to be facilitated by an increased urge for movement and physical restlessness, particular to AN. The increased urge to move would reflect spontaneous daily activity, the energy expended for everything that is not sleeping, eating, or voluntary exercise. Our hypothesis is that the starvation-induced increased urge to move and restlessness may promote the development of AN. Reversal of the fasting state, by either high caloric food or by leptin administration, would be expected to reduce restlessness and the increased urge to move along with improvement in other symptoms in AN. This review explores the idea that such restless activation in AN, in itself and through accelerating body weight loss, might foster the integration of the starving body into the self-concept by (1) enhancing the person’s sense of self-control and sense of achievement and (2) through invigorating proprioception and through intensifying the perception of the changing body shape. (3) Tentative evidence from studies piloting leptin administration in chronic AN patients which support this hypothesis is reviewed. The findings show that short term administration of high doses of leptin indeed mitigated depressive feelings, inner tension, intrusive thoughts of food, and the increased urge to be physically active, easing the way to recovery, yet had little influence on the patients’ personal commitment to remain at a low weight. Full recovery then requires resolution of the individuals’ personal unresolved psychological conflicts through psychotherapy and frequently needs specialized treatment approaches to address psychiatric co-morbidities. AN might be conceptualized as a hereditary form of starvation resistance, facilitated by the effects of starvation on fitness allowing for an exceptionally intense personal commitment to perpetuate food restriction.

## Introduction

AN, a disorder of long-term voluntary restricted food intake resulting in recalcitrant pathological weight loss in female adolescents is considered a psycho-biological disorder. Predominantly, AN is, but must not be, a psychiatric disorder. Emotional disturbances and psychiatric co-morbidity vary from person to person and young patients often recover after short-term treatment and lead a normal life (Halvorsen and Heyerdahl, 2006). Psychological features include subjective personal risk factors such as a critical self-image (Steinhausen and Vollrath, 1993) along with distinct personality features depending on the subtype (Casper et al., 1980). In the restricting form, shyness, a sense of being overlooked and not appreciated, may lead to the appropriation of caloric restriction as a way to prove themselves worthy. A family history of psychiatric illness bears on illness severity (Strober et al., 1990).

On the biological side, the significant physiological adjustments to caloric deficiency of virtually every organ system, including the brain, none different from those in simple starvation, have not revealed particular mechanisms which could account for the excessive weight loss observed in AN. On the genetic side, anorexia nervosa (AN) is considered a complex, multifactorial heritable condition with possibly rare and common genetic and environmental determinants (Cui et al., 2013; Boyle et al., 2017; Lee et al., 2019; Watson et al., 2019). Heritability estimates in twin studies consistently support a genetic basis in anorexia nervosa, with the range of 48–74% (Holland et al., 1984; Klump et al., 2001; Kortegaard et al., 2001) Recently, the Anorexia Nervosa Genetics Initiative was launched to increase sample sizes for pinpointing genetic risk factors (Yilmaz et al., 2020).

At this point in research, therefore, reliance on clinically observable signs remains a useful heuristic method to identify aberrant processes in AN.

The name “anorexia nervosa’ translates to ‘nervous lack of appetite.” AN patients, however, endorse hunger feelings (Garfinkel, 1974) and they report conscious and intrusive thoughts and dreams of food (Casper and Davis, 1977), similar to undernourished individuals.

Surprisingly, individuals with AN tend to divulge few signs of distress, asserting that they do not feel changed and therefore oppose treatment for underweight. Adolescent girls with AN tend to live seemingly normal lives, they rise early in the morning, refreshed without complaints of fatigue to engage in their daily activities (Casper, 1990). They continue to function in school, even at their lowest body weight, when cachexia undermines muscle strength (Casper, 2020). This ability to pursue an active, seemingly normal, life, as much as a not infrequent tendency toward excessive physical activity in AN has been commented on since its earliest descriptions (Gull, 1874; Janet, 1903; Bell, 1985), contrasting it to the lassitude and weariness associated with advanced simple starvation (Guetzkow and Bowman, 1946; Keys et al., 1950).

We outline here experimental and clinical evidence that healthy humans undergoing starvation experience a slowing of movements and tend to avoid voluntary exercise. We then present studies that found that total energy expenditure and physical activity levels in AN did not differ from normal controls. Next, we address the data that physical restlessness and an increased urge for movement at low weights are common symptoms in AN. Bearing in mind that AN is a human and personally motivated disorder, we will examine the contribution of a critical self-image and lack of self-esteem to the pursuit of caloric restriction. We suggest that, not only the weight loss, but also the possibly energizing physical sensations (Casper, 2020) associated with an increased urge for movement and physical restlessness may strengthen the patient’s self-control and subjective vitality (Ryan and Frederick, 1997), as well as motivation to use dieting to improve the self-image. Next, we discuss experimental studies examining the body image in AN and argue that restless body sensations might contribute to the tendency to overestimate body dimensions and to the selective deficient subjective awareness of the wasted body. Lastly, if the increased urge to move constitutes an integral component of the anorectic symptom constellation, then a reversal of the negative metabolic balance would be expected to reduce the increased urge to move. We will review pilot studies showing that leptin (Metreleptin) administration in high doses signaling a high energy status was associated with significant symptomatic improvement in critical, but not all symptoms in chronic AN patients (Milos et al., 2020; Antel et al., 2021).

## Diminished Physical Activity and Inertia in Experimental Starvation Contrast With Reports of an Increased Urge for Movement and Physical Restlessness at the Lowest Weight in AN

Physical activity levels in humans are complex and influenced by psychological, social, multiple genetic and environmental factors (Carlsson et al., 2006; Stubbe et al., 2006; Duncan et al., 2008). Physical activity forms part of the energy balance feedback system that homeo-statically controls body weight. Supporting data have come from overfeeding studies in healthy subjects which showed compensatory changes increasing spontaneous activity or non-exercise activity thermogenesis (NEAT) to dissipate excess energy (Leibel et al., 1995) and from underfeeding studies (Guetzkow and Bowman, 1946; Keys et al., 1950; Consolazio et al., 1967; Heilbronn et al., 2006; Martin et al., 2007; Redman et al., 2008) which show a reduction in physical activity levels to conserve energy.

Fasting or energy depletion triggers compensatory metabolic, endocrine and behavioral responses to limit energy expenditure and stimulate food intake. Measurements in experimental starvation invariably show a “slowing of voluntary movements and curtailment of self-initiated spontaneous activity” and reduced ‘drive for activity’ measurements. After achieving a 25% weight loss in 6 months, 94% of the men reported “unsteadiness and uncertainty of footing while walking” (Keys et al., 1950). The men also experienced anxious-depressive symptoms, social withdrawal, obsession with food, and sexual dysfunction. In retrospect, these men remembered leaden lethargy and exhaustion (Eckert et al., 2018), all feeling states not reported by AN patients at a greater weight loss. These findings raise the question whether the persistence of normal physical activity in AN is atypical and possibly pathognomonic.

In the past two decades, numerous investigators have addressed primarily the phenomenon of excessive exercise and compulsion to exercise in AN (Davis et al., 1994; Holtkamp et al., 2004; Davis and Kaptein, 2006; El Ghoch et al., 2013; Keyes et al., 2015; Rizk et al., 2015; Schlegl et al., 2018).

Many investigators have attributed the pursuit of exercise in AN to psychological mechanisms suggesting that physical activity is a deliberate attempt to “burn” calories in pursuit of thinness (Davis et al., 1997) or that physical activity is a way of coping with negative affect (Davis et al., 1999; Holtkamp et al., 2006). These factors undoubtedly play a role both in AN and in healthy dieting individuals, yet, exercise normally is not associated with pathological weight loss.

Aside from the tendency to excessive exercise, spontaneous or non-exercise activity, such as standing and walking in AN has been found comparable to non-exercise activity in healthy subjects (Keyes et al., 2015). Assessments through questionnaires and through recordings of wearable devices found that daily activity levels measured as steps or as body movements did not distinguish AN patients from healthy age- and sex-matched controls (Bouten et al., 1996; Van Marken Lichtenbelt et al., 1997; Harris et al., 2008; Hechler et al., 2008; Bratland-Sanda et al., 2010a,b; Hofmann et al., 2014; Keyes et al., 2015).

Another rather neglected observable symptom in AN patients is their greater restlessness, confirmed by Belak et al. (2017) in leg movements of seated AN patients, wearing a shoe-based monitor. Clinical ratings, of adolescent AN patients (Holtkamp et al., 2006) found an inverse relationship between subjective and accelerator measures of physical activity as well as self-rated motor and inner restlessness with plasma leptin levels, pointing to the degree of undernutrition as a crucial factor for maintaining motor activity.

The ability to remain “normally” active in AN raises the question to what extent intrinsic AN-specific biological mechanisms triggered by caloric restriction contribute to physical restlessness? Are AN patients spared the full impact of the low energy levels and fatigue typically reported in starvation states?

In an exploratory study, 83 adolescent patients with acute AN, restricting type, were systematically asked on hospital admission, whether or not they experienced an increased urge to move and physical restlessness at their lowest weight (Casper et al., 2020). Nearly 90% of patients reported either, an increased urge for movement and/or physical restlessness. Two thirds endorsed feeling mentally alert and being able to concentrate. The increased urge to move emerged as a physiological variable related to the degree of weight loss, to feeling active, to movement despite feeling tired, and to exercise intensity and compulsive exercise before hospital admission, but was not related to the severity of the eating disorder or the severity of psychiatric symptoms. Unexpectedly, the increased urge to move coexisted side by side with high levels of physical fatigue and low energy, typical signs of starvation. Physical restlessness, endorsed by 82%, was associated with the degree of weight loss and anxiety levels. Mental restlessness emerged as a disease severity variable indicating widespread strong correlations with signs of starvation and with eating disorder and psychiatric symptomatology. It would be interesting to explore further, how much AN patients are aware of an increased urge to move.

We can infer from these observations that adolescent patients with acute AN at their greatest weight loss feel compelled to move and physically restless despite experiencing fatigue and feeling tired. Since such restless activation has not been reported to exist at the lowest weight in experimental starvation, it might point to dysfunctional physiological adaptations in energy regulating pathways in AN (Casper, 2016).

*Hypothesis 1*: The ability of AN patients to maintain an active life style and regular daily activities may not only strengthen the resolve to limit food intake, but also impart a sense of self-control and a sense of achievement and thereby improve self-esteem.

AN is a personalized disorder. Patients seize control over the starvation process for self-improvement, to the extent that a reciprocal relationship develops between successful weight control and positive emotions. Personal satisfaction over weight loss is also an important reward in normal dieters. In a healthy person, unlike in AN, the physical discomforts and fatigue of chronic undernutrition and the presence of other rewarding activities, maintaining friendships, enjoying music, taking dance classes, seem to undermine the single-minded pursuit of continuing a fasting regime.

The concepts of self-image and self-esteem refer to a person’s basic evaluative feelings about her/himself. Low self-esteem has been described in a number of studies as a characteristic attribute of AN patients (Silverstone, 1990) and has been found to be a predictor of co-morbidity (Karatzias et al., 2010) and poor treatment outcome (Halvorsen and Heyerdahl, 2006). The tendency for self-reliance in teenage AN patients, only 17% experienced friendships as positive (Datta et al., 2021), heightens the personal impact of this solipsistic interaction.

An American and a European study (Casper et al., 1981; Steinhausen and Vollrath, 1993) found nearly identical responses on the Offer self- image questionnaire reflecting a poor self-image and body image in teenage hospitalized AN patients compared to age-matched healthy controls. Findings that the self-concept and body image improve with treatment, but remain unchanged without treatment, highlight the importance of self/body concept disturbances as a core psychological problem in severe AN. This link between self-esteem and body esteem in women with eating disorders, was not found in healthy women (Mendelson et al., 2002). Indeed, internal rewards, the wish for a sense of self-respect, power and independence raising self-esteem have been described to play a decisive role in the fixation on weight loss in AN (Garner and Bemis, 1982; Serpell et al., 1999; Brockmeyer et al., 2013).

We suggest that the propelling force of the increased urge to move and physical restlessness in AN which counteracts the physical discomforts of chronic undernutrition tends to strengthen the resolve of AN patients to extend caloric restriction. Successful reduction in food intake and the ensuing weight loss then enhance the sense of personal efficiency and self-control. Nonetheless, the gradually increasing fear of weight gain in AN betrays some awareness that patients are cognizant that caloric restriction is a spurious and in the long-term dangerous solution to low self-esteem.

*Hypothesis 2*: The increased urge to move and physical restlessness enhance proprioception and body awareness and thereby counterbalance awareness of the emaciated body and may contribute to body size overestimation in AN.

Altered body perceptions in AN have been the subject of comment and investigation ever since Lasègue (1873) quoted an AN patient as saying, − in response to the comment that her amount of nutrition could not support a toddler,- that she is “neither changed nor thinner, moreover she has never refused a task or suffered fatigue.” This patient‘s response, insisting on sameness despite her emaciation, reflects the contentment with the body’s form in AN. In other words, it reflects an insufficient awareness of the increasingly lean body contours in AN (Bruch, 1962). In experimental or simple starvation body image disturbances have not been reported (Keys et al., 1950; Eckert et al., 2018).

We propose here that physical restlessness and an increased urge to move during weight loss not only convey a welcome sense of lightness, but also intensify proprioception and in this way integrate the bodily changes in AN. This hypothesis is supported by findings in healthy populations where movement through physical exercise contributes to positive body-and self-evaluations (Guinn et al., 1997; Hausenblas and Fallon, 2006; Korn et al., 2013). Movement in exercise also modifies proprioception (Bhanpuri et al., 2013; Proske and Allen, 2019). Proprioception is defined here as the sensation of body position and movement, the senses of tension and effort and the sense of balance through mechanically sensitive receptors, with muscle spindles as the principal proprioceptors (Longo and Haggard, 2010; Proske and Gandevia, 2012). Conversely, frequent complaints of unpleasant feelings of heaviness voiced by AN patients on weight gain are associated with a reduction in restlessness and the desire to move (Holtkamp et al., 2003a).

The concept of central representations of the body was first described by Head and Holmes (1911) based on selective loss of sensations after injury. Schilder (1950) used the term “body image” as a construct of the mental representation of the body encompassing imaginative, perceptual, affective and cognitive components. Schilder (1950) views the body image as “the picture of our own body as we perceive it and as we imagine it. It does not merely consist of perception in the common sense, but it comprises elements of representations and thoughts.”

The precise definition of the body image and the body schema and whether they ought to be considered different entities are still under discussion (Tuthill and Azim, 2018). Whereas the body image as the cognitive representation of the body is thought of as being based on stored life experience and to underlie perceptual judgment, the body schema is believed to depend on ongoing proprioceptive input and concerned with body movement. The term ‘body image’ as used in AN research is rarely defined, but seems to be an amalgamation of both definitions. Distortions in the topographical map of the body or in sensorimotor perceptions have not been reported in AN (Phillipou et al., 2016).

The DSM V diagnostic criteria for AN (DSM-V, 2013) define body image changes functionally, as “a disturbance in the way in which one’s body weight or shape is experienced, undue influence of body weight or shape on self-evaluation, or persistent lack of recognition of the seriousness of the current low body weight.”

It is important to remember that not all AN patients initially aim to lose weight. In particular, younger patients often renounce food or reduce their food intake due to maturity fears or out of moral or ethical concerns. Nonetheless, ultimately, AN patients fulfill all three DSM-V criteria for body image changes.

Some researchers (Gutierrez and Carrera, 2020) have argued that body image disturbances in AN are merely epiphenomena of the low body weight. This proposition may be correct, but it is difficult to ascertain, considering the absence of such extreme low weights without disease.

Deficient recognition of the bony contours and lack of alarm over the implications and risks associated with extreme fasting and emaciation are core symptoms which distinguish AN from weight loss for other reasons. A case illustrates this lack of awareness. The only patient ever to seek treatment on her own in our program was a 20-year old young woman, who called, sounding panic-stricken. She described how that very morning a rear view mirror had by chance projected skeletal shoulders which she thought to belong to another person standing behind her. Frightened, she turned around and realized she had seen her own back. At that moment she realized for the first time the extent of her emaciation. The patient immediately placed the call for a consultation, agreed to hospitalization, cooperated in treatment and fully recovered.

This awareness deficit of the extent of the emaciation in AN seems to be partial to the life-endangering consequences of the low body weight. Risks to health and life from energy deficiency are kept out of awareness or minimized. Unless asked, patients do not mention physical discomfort from constipation or from feeling constantly cold. Surprisingly, awareness of the desired thinness seems to be intact. Excessive thinness is acknowledged through behavior, such as buying size 2 clothes, displaying the bony body in scant clothing in the summer, or the habit of secret mirror gazing and admiring the emaciated body in private.

Numerous PET studies have, so far unsuccessfully, attempted to localize a central defect in body image representations in AN. They report altered structures and networks linked to measures of starvation (Lankenau et al., 1985; Krieg et al., 1989; Favaro et al., 2012; Via et al., 2014) in line with early reports of significant macroscopic brain changes in AN (Enzmann and Lane, 1977), all changes reversible with weight recovery. Normative comparisons do not exist, because healthy normal weight individuals do not suffer the extreme weight loss of AN patients.

The first criterion of DSM V (DSM-V, 2013) “disturbance in the way in which one’s body weight or shape is experienced “as well as disturbances in estimating body part boundaries have been supported by empirical findings. A number of studies (Slade and Russell, 1973; Crisp and Kalucy, 1974; Garner et al., 1976; Casper et al., 1979; Touyz et al., 1984; Sachdev et al., 2008; Guardia et al., 2012; Verbe et al., 2021) confirm varying and selective degrees of body size overestimation in AN, in particular for the waist, chest and body depth by 14–30%. Their specificity remains uncertain, because the studies found similar degrees of body size overestimation in healthy age-matched women, indicating fluidity in the assessment of body size. In AN, importantly, the degree of body size overestimation was found to be associated with illness severity, with lack of recognition of the seriousness of the illness, the amount of weight loss, and prognosis and treatment response (Casper et al., 1979). The more accurate the body size estimate, the better the long-term outcome. Thus, body size overestimation, albeit quite common in contemporary healthy females, appears to be a marker of illness severity in AN.

**Figure 1:**
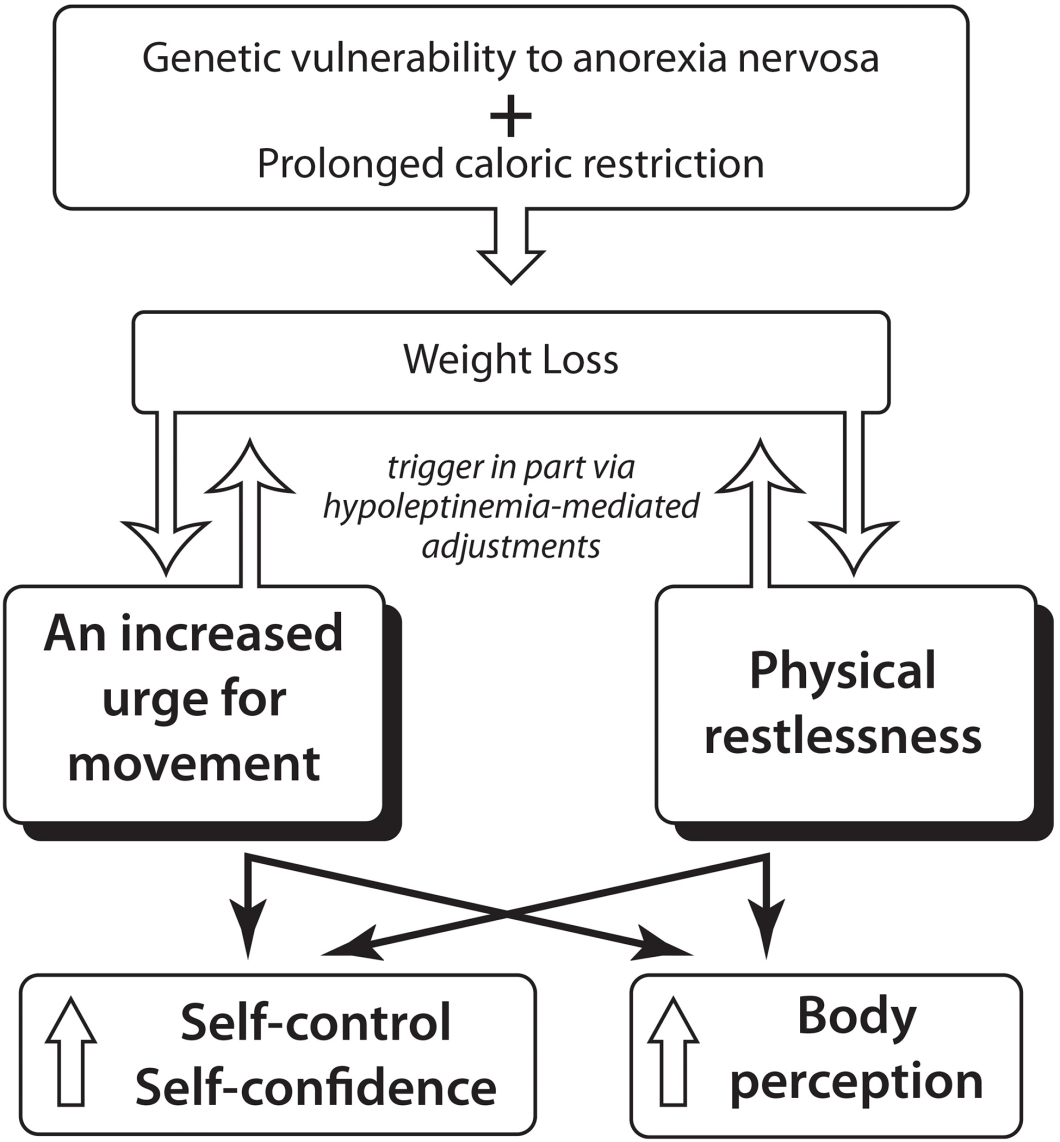
Hypothesis: An increased urge for movement and physical restlessness in Anorexia Nervosa might foster the integration of the starved thin body into the self-concept through enhancing the person’s sense of self-control and might through intensifying body perception influence body size estimation.

Incomplete consciousness of the bodily changes with severe weight loss and body size overestimation likely contributes to the fear of weight gain which paradoxically gets stronger with increasing weight loss. Alternately, treatment leading to a significant weight increase lessens this fear of weight gain (Calugi et al., 2018; Figure 1).

*Hypothesis 3*: As a component of the starvation-induced anorectic symptom complex associated with hypo-leptinemia, the increased urge to move ought to improve along with other symptoms in AN through treatment with leptin (Metreleptin) which produces a high energy metabolic condition.

Leptin is a regulatory hormone secreted into the bloodstream by white adipose tissue. Leptin belongs to the family of class 1A cytokine receptors in brain and the periphery to regulate energy balance, neuroendocrine function, immune function and various metabolic pathways, including growth hormone signaling, insulin sensitivity, and lipogenesis (Salbe et al., 1997; Allison and Myers, 2014). Leptin is also expressed in a variety of other tissues, including the placenta, ovaries, mammary epithelium, bone marrow, and lymphoid tissues (Margetic et al., 2002).

Leptin binds to leptin receptors located throughout the central nervous system and peripheral tissues (Mantzoros et al., 2011), including skeletal muscle (Guerra et al., 2007). Prolactin releasing peptide containing neurons sensitive to leptin in the dorsomedial hypothalamic nucleus seem to mediate thermogenesis and increase energy expenditure (Dodd et al., 2014).

Leptin is a sensor for the adaptation to starvation and signals a state of nutrient deficiency and fat loss. Levels of leptin are directly proportional to adipose tissues and decrease with undernutrition (Blum et al., 1997). The leptin regulatory system is profoundly affected by the metabolism of starvation in AN (Ahima et al., 1996; Hebebrand et al., 1997; Chan et al., 2003; Mars et al., 2006).

The decrease in leptin levels during fasting mediates the suppression of reproductive, thyroid, and growth hormones and the elevation in glucocorticoid levels, in addition to stimulating food intake and limiting energy expenditure (Ahima et al., 1996, 2000).

Transport of leptin to the brain is reduced by fasting (Kastin et al., 2000). In a genome-wide meta-analysis, Kilpeläinen (2016) uncovered several loci associated with circulating leptin levels and Peters et al. (2021) recently reported a correlation between a genetic predisposition to low leptin levels and risk for AN in females.

Depending on the degree and severity of the caloric deficit and weight loss, leptin falls to very low, sometimes undetectable levels in AN (Grinspoon et al., 1996; Hebebrand et al., 1997) and increases significantly with weight gain (Eckert et al., 1998). In fact, leptin plasma levels below 2 μg/l have been found to have high specificity and high sensitivity for acute AN (Focker et al., 2011). Mantzoros et al. (1997) report a significantly higher CSF to plasma leptin ratio in AN patients compared to healthy controls. Leptin plasma levels have been found to be negatively associated with excessive physical activity in acute, but not in recovered, AN patients (Hebebrand et al., 2003; Holtkamp et al., 2003b; Ehrlich et al., 2009).

Regarding leptin parameters in normal populations, light, but not moderate and vigorous, physical activity assessed by accelerometer for 4 weeks were associated with the Q223R polymorphism in the leptin receptor gene of female, but not male, Japanese adults, suggesting a genetic influence on spontaneous physical activity (Murakami et al., 2014). Leptin receptor variants have also been associated with habitual physical activity assessed by self-report in European-derived volunteers (Walsh et al., 2012) and polymorphisms in the dopamine D2 receptor gene have been linked to physical activity levels among white women (Simonen et al., 2003). Considering the regression to prepubertal status in AN, correlations between plasma leptin concentrations with total energy expenditure and physical activity in five-year old children and 8-year-old healthy girls with steps measured by pedometer, respectively, are noteworthy findings (Salbe et al., 1997; Romon et al., 2004). In healthy adolescent girls vigorous physical activity measured by accelerometer over 7 days, increasing the energy expenditure, was negatively associated with leptin concentrations (Jiménez-Pavón et al., 2012). In male soldiers serum leptin decreased to a third of normal levels along with energy deficiency induced by 4 weeks of strenuous military training (Gomez-Merino et al., 2002). Similarly, exercise addiction in young men was found associated with lower plasma leptin levels (Lichtenstein et al., 2015).

In rodents, deletion of the leptin receptor in dopamine neurons induced anxiety like behavior (Liu et al., 2011), while intraperitoneal injection of leptin increased exploration and social behaviors (Liu et al., 2010). Systemic leptin treatment produced antidepressant effects *via* limbic structures (Lu et al., 2006).

The metabolic effects of systemic leptin administration have been tested in congenital leptin deficiency states (Farooqi et al., 2001), lipodystrophy and dysfunction of the hypothalamic–pituitary-gonadal axis. Leptin receptor-expressing pericytes have been found to mediate vessel permeability and promote leptin brain uptake (Butiaeva et al., 2021). Low plasma leptin levels prompted Welt et al. (2004) to treat patients with hypothalamic amenorrhea due to underweight or strenuous exercise restoring ovulatory menstrual cycles in 3 of eight patients, without apparent adverse effects. Chou et al. (2011) conducted a randomized, placebo-controlled trial with human recombinant leptin for 36 weeks. Seven of 10 patients with hypothalamic amenorrhea receiving metreleptin therapy developed menstruation during the course of the study, and two of nine subjects on placebo developed menstruation. In strenuously exercising lean young women with hypothalamic amenorrhea, metreleptin treatment over 1–2 years increased bone mineral density and content in the lumbar spine in the presence of moderate improvement in other endocrine and metabolic parameters (Sienkiewicz et al., 2011).

**Figure 2:**
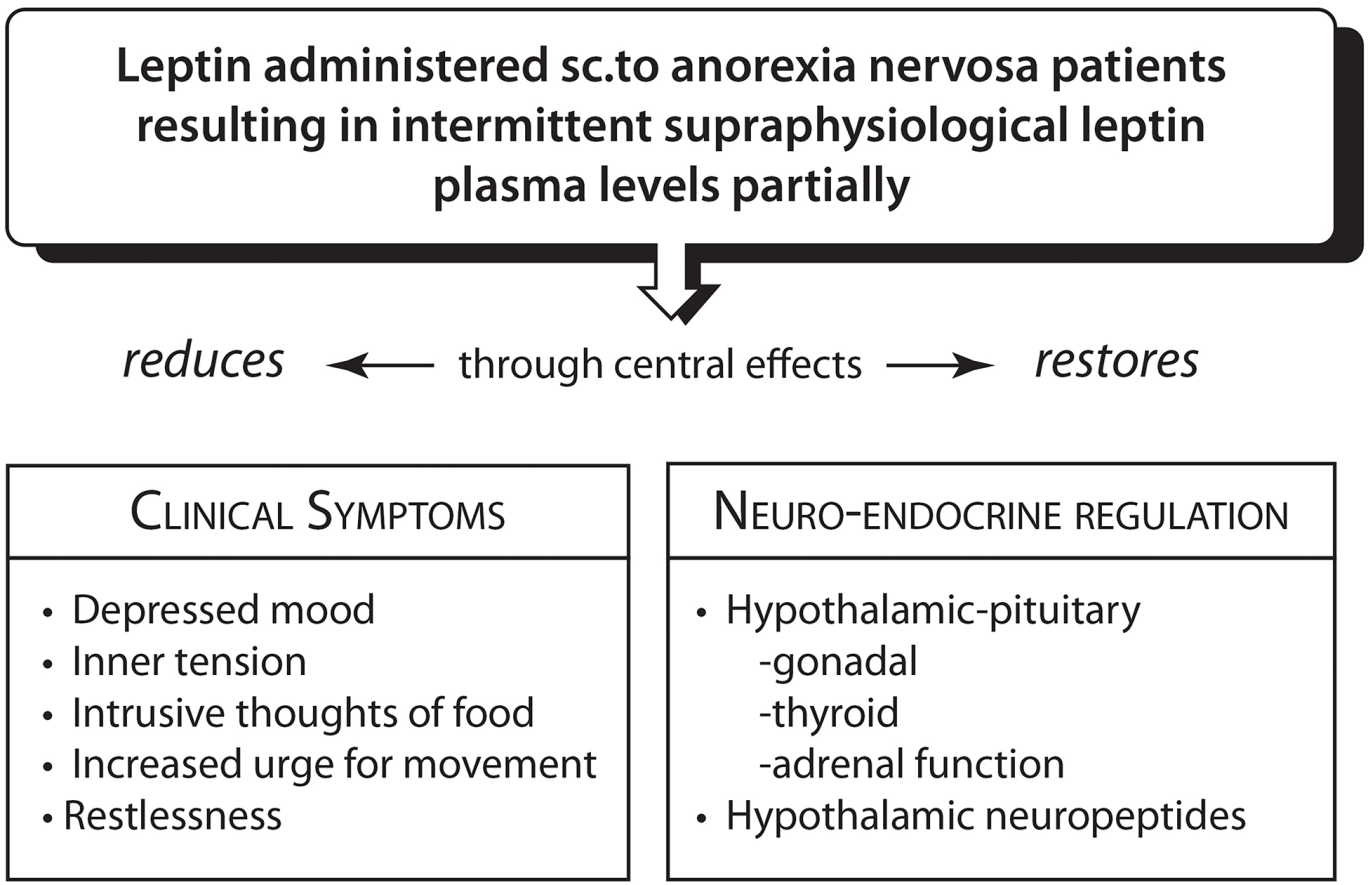
Reversal of hypoleptinemia-mediated adjustments to prolonged severe caloric restriction in anorexia nervosa by Metreleptin (Ahima et al., 1996; Rosenbaum et al., 2002; Welt et al., 2004; Lu et al., 2006; Mantzoros et al., 2011; Licinio et al., 2014; Wang et al., 2015; Chrysafi et al., 2020; Milos et al., 2020; Antel et al., 2021).

In response to the long suffering of chronic AN patients and in view of the reported safety profile of Metreleptin, Hebebrand and co-workers (Milos et al., 2020; Antel et al., 2021) designed a pilot study to administer short-term subcutaneous Metreleptin to four patients with severe chronic AN. The daily doses were higher than those in hypothalamic amenorrhea, but given for much shorter periods, between 7 and 22 days, raising leptin plasma concentrations from an extreme low to high levels. Within 24 to 48 h, self-and observer reports documented rapid improvement in an ensemble of AN symptoms: depressed mood, urge for activity, inner tension, repetitive thought of food in ¾ patients (Milos et al., 2020, Antel et al., 2021). The symptom reduction suggests wide-ranging central effects of leptin (Chan et al., 2003; Chan and Mantzoros, 2005). Metreleptin administration which produced high leptin plasma levels within 2 to 7 h, was also associated with greater insight into the condition, greater sociability and in a 16 year old male patient produced normalization of hypogonadotropic hypogonadism. Restlessness was reduced, patients were able to nap and sit still for longer periods. Cessation of leptin administration led to reemergence of symptoms after 5–8 days. Despite reporting “a more realistic assessment of body shape and weight” (Milos et al., 2020), the patients’ personal investment in maintaining a low weight showed modest changes and required long-term skilled psychological treatment to encourage weight recovery (Hebebrand et al., 2021). These observations provide tentative support that an acute reversal of the negative metabolic balance through very high peripheral and central circulating leptin levels which are associated with endocrine changes (Rosenbaum et al., 2002; Antel et al., 2021) swiftly, albeit partly, mitigate the anorectic symptom constellation including the maladaptive urge for movement and physical restlessness in AN (Figure 2).

## Conclusion

There is agreement that the etiology of AN involves a complex interplay between polygenic risk variants and specific environmental triggers, principally prolonged caloric restriction.

Taking up Nurse’s (2021) admonition that” biology must generate ideas as well as data,” we provide here reasonable evidence for the proposition that in individuals genetically vulnerable to AN, restlessness and an increased urge for movement triggered by the negative metabolic balance, and varying in intensity, may directly through forestalling the full impact of starvation and indirectly through movement effects promote the personal decision to continue caloric restriction and hence have a bearing in the development of AN. As AN develops, such “restless activation” not only counteracts the fatigue and lower energy level of the starvation state, but also accelerates weight loss and thereby strengthens personal resolve and the sense of control. Through heightening proprioception, physical restlessness and an increased urge to move might contribute to appropriating the reduced body contours into the self-concept and contribute to neglecting the life-endangering consequences of the severe loss of body weight. High circulating leptin concentrations signaling a positive metabolic balance associated with endocrine changes remarkably swiftly improve several anorectic symptoms, including the physical restlessness and the increased urge to move. AN might be conceptualized as a hereditary form of starvation resistance, facilitated by the effects of starvation on fitness allowing for an exceptionally intense personal commitment to perpetuate food restriction. The suggested hypotheses are testable and could be integrated into the design of future double blind placebo-controlled studies of leptin administration as a therapeutic strategy in AN.

## Author Contributions

The author confirms being the sole contributor of this work and has approved it for publication.

## Conflict of Interest

The author declares that the research was conducted in the absence of any commercial or financial relationships that could be construed as a potential conflict of interest.

## Publisher’s Note

All claims expressed in this article are solely those of the authors and do not necessarily represent those of their affiliated organizations, or those of the publisher, the editors and the reviewers. Any product that may be evaluated in this article, or claim that may be made by its manufacturer, is not guaranteed or endorsed by the publisher.
